# MiR-133b Is Down-Regulated in Human Osteosarcoma and Inhibits Osteosarcoma Cells Proliferation, Migration and Invasion, and Promotes Apoptosis

**DOI:** 10.1371/journal.pone.0083571

**Published:** 2013-12-31

**Authors:** Huafu Zhao, Mei Li, Lihua Li, Xiaoming Yang, Guobo Lan, Yu Zhang

**Affiliations:** 1 Department of Medical Research, Liu Hua Qiao Hospital, Guangzhou, Guangdong Province, China; 2 Department of Orthopedic Surgery, Liu Hua Qiao Hospital, Guangzhou, Guangdong Province, China; University of Kentucky College of Medicine, United States of America

## Abstract

MicroRNAs (miRNAs) decrease the expression of specific target oncogenes or tumor suppressor genes and thereby play crucial roles in tumorigenesis and tumor growth. To date, the potential miRNAs regulating osteosarcoma growth and progression are not fully identified yet. In this study, the miRNA microarray assay and hierarchical clustering analysis were performed in human osteosarcoma samples. In comparison with normal human skeletal muscle, 43 miRNAs were significantly differentially expressed in human osteosarcomas (fold change ≥2 and *p*≤0.05). Among these miRNAs, miR-133a and miR-133b expression was decreased by 135 folds and 47 folds respectively and the decreased expression was confirmed in both frozen and paraffin-embedded osteosarcoma samples. The miR-133b precursor expression vector was then transfected into osteosarcoma cell lines U2-OS and MG-63, and the stable transfectants were selected by puromycin. We found that stable over-expression of miR-133b in osteosarcoma cell lines U2-OS and MG-63 inhibited cell proliferation, invasion and migration, and induced apoptosis. Further, over-expression of miR-133b decreased the expression of predicted target genes BCL2L2, MCL-1, IGF1R and MET, as well as the expression of phospho-Akt and FAK. This study provides a new insight into miRNAs dysregulation in osteosarcoma, and indicates that miR-133b may play as a tumor suppressor gene in osteosarcoma.

## Introduction

Osteosarcoma (OS) is an aggressive bone tumor characterized by malignant osteoid production and osteoblastic differentiation. It is an infrequent but the most common and destructive primary bone tumor in children and adolescent. In the past decades, the surgical resection therapy resulted in poor prognosis of OS patients. With the application of neoadjuvant chemotherapy using cisplatin, doxorubicin, ifosfamide and methotrexate in osteosarcoma, the 5-year survival rate has increased to approximate 50%–80% [1], [2]. To date, however, the molecular pathogenesis and etiology of osteosarcoma are still not clearly elucidated. MicroRNA (miRNA or miR), a short conserved non-coding RNA with 22∼24 nucleotides long, is believed to be a promising diagnostic and prognostic tool of malignant neoplasm [3]. By binding to the complementary target mRNA, miRNAs lead to mRNA degradation or preventing mRNA from being translated. Therefore, miRNAs regulate target genes expression at post-transcriptional level. Over-expression of miRNAs usually gives rise to the deceased expression of target genes. Evidence showes that 98 miRNAs locate at fragile sites and genomic regions involved in cancer, indicating that miRNAs are tightly interrelated with tumorigenesis and tumor progression [4]. By mediating the expression of oncogenes or tumor suppressors, miRNAs play a critical role in tumor growth, progression, metastasis, and drug resistance [5], [6].

MiR-133b is a member of miR-133 family and known as a muscle-specific miRNA, mediating myoblasts proliferation and differentiation [7]. However, although studies including expression restoration and antisense specific knockdown of miR-133b have unraveled many aspects of its function and targets in muscle development, little evidence reveal its role in the development of sarcoma, especially in osteosarcoma. Recently, miR-133b is also identified in muscle-derived sarcomas [8]. In addition, miR-133b expression is decreased in gastric cancer, colorectal cancer, bladder cancer, prostate cancer and lung cancer, indicating that miR-133b plays an important role in tumorigenesis and cancer progression [9]–[13]. However, the expression and functional roles of miR-133b in osteosarcoma are unknown yet.

In this study, miRNA expression profiles of human osteosarcoma samples were compared with those of adjacent normal skeletal muscle. A set of miRNAs were identified to be significantly down-regulated or up-regulated in osteosarcomas, indicating that they may be the potential tumor suppressor genes or oncogenes. Validated by quantitative real-time PCR (qRT-PCR), miR-133b expression was confirmed to be significantly decreased in both frozen and paraffin-embedded osteosarcoma samples. Over-expression of miR-133b in osteosarcoma cell lines U2-OS and MG-63 led to the inhibition of cell proliferation, migration, invasion through decreasing the expression of IGF1R, MET, phospho-Akt and FAK. In addition, miR-133b over-expression increased apoptosis of OS cells through down-regulation of anti-apoptotic molecules BCL2L2, MCL-1 expression.

## Materials and Methods

### Reagents and antibodies

Monoclonal anti-BCL2L2 (#2724), anti-MCL-1 (#5433), anti-IGF1Rβ (#9750), anti-MET (#8198), anti-phospho-Akt (Ser473) (#9271), anti-Akt (#9272), anti-PTEN (#9552), anti-FAK (#3285), anti-β-Tubulin (#2146) and horseradish peroxides (HRP)-conjugated secondary antibody (#7074) were purchased from Cell Signaling Technology.

### Tumor specimens

Primary osteosarcoma tumor samples were obtained from 23 patients with average age of 20 years (range from 11 to 39 years) in Liu Hua Qiao Hospital (Table 1). About two-thirds of patients were males, and more than 90% of osteosarcomas occurred in extremities. 18 of 23 tumor samples were obtained from formalin-fixed paraffin-embedded blocks, whereas the other five samples used in the microRNA microarray assay were stored in the liquid nitrogen. All tumor samples were high-grade osteosarcomas with either stage IIA or IIB in Enneking system, except for one tumor with stage III. The subtypes of osteosarcoma included conventional (34.8%, 8 of 23), osteoblastoma-like (21.7%, 5 of 23), chondroblastoma-like (17.4%, 4 of 23), fibroblastic, fibrohistiocytic, and other non-conventional OS: telangiectatic, periosteal, intraosseous well-differentiated and high-grade surface. Three human normal muscle from patients OS-1, OS-4 and OS-5 and two formalin-fixed paraffin-embedded normal bones from patients P-OS-4 and P-OS-10 were used as the negative controls. The identities of all tumor, normal muscle and bone samples were confirmed by an experienced pathologist. This study was approved by the Research Ethics Committee of Liu Hua Qiao Hospital. All patients were given written informed consent.

**Table 1:** Clinical characteristics of patients with high-grade osteosarcoma.

No.	Age(years)/Gender	Subtypes	Enneking stages	Sites	Follow-up
OS-1	12/Female	Chondroblastoma-like	IIA	Femur	Alive
OS-2	16/Female	Osteoblastoma-like	IIB	Femur	Withdraw
OS-3	23/Female	Conventional	IIA	Tibia	Alive
OS-4	20/Male	Chondroblastoma-like	IIB	Fibula	Alive
OS-5	30/Male	Telangiectatic	IIB	Femur	Dead
P-OS-1	11/Female	Osteoblastoma-like	IIA	Tibia	Alive
P-OS-2	16/Male	Conventional	IIA	Femur	Withdraw
P-OS-3	20/Male	Osteoblastoma-like	IIB	Femur	Withdraw
P-OS-4	21/Female	Periosteal	IIA	Femur	Alive
P-OS-5	16/Male	Conventional	III	Thoracic wall	Withdraw
P-OS-6	20/Male	Fibroblastic	IIA	Tibia	Dead
P-OS-7	25/Male	Chondroblastoma-like	IIB	Pubis	Withdraw
P-OS-8	17/Male	Chondroblastoma-like	IIB	Femur	Dead
P-OS-9	15/Male	Conventional	IIB	Femur, Tibia	Dead
P-OS-10	28/Female	Fibrohistiocytic	IIB	Femur	Dead
P-OS-11	14/Male	Conventional	IIB	Femur	Dead
P-OS-12	15/Female	Conventional	IIA	Femur	Withdraw
P-OS-13	39/Male	Conventional	IIB	Femur	Alive
P-OS-14	21/Male	Osteoblastoma-like	IIA	Femur	Withdraw
P-OS-15	20/Male	Intraosseous well-differentiated	IIA	Femur	Withdraw
P-OS-16	24/Female	High-grade surface	IIB	Tibia	Withdraw
P-OS-17	21/Male	Conventional	IIB	Humerus	Withdraw
P-OS-18	14/Female	Osteoblastoma-like	IIA	Femur	Dead

### Cell culture

Human osteosarcoma cell lines U2-OS and MG-63 were purchased from the Type Culture Collection of the Chinese Academy of Sciences. U2-OS and MG-63 cells were cultured in McCoy's 5A medium and Minimum Essential Medium (MEM) supplemented with 10% fetal bovine serum (FBS), respectively. Cells were incubated at 37°C in 5% CO_2_ atmosphere and passaged every two to three days.

### Isolation of miRNAs

According to the manufacture's protocol, total RNAs including miRNAs in frozen osteosarcoma and muscle samples were isolated using miRNeasy Mini Kit (Qiagen), while miRNAs of formalin-fixed paraffin-embedded samples were extracted by FFPE miRNeasy Kit (Qiagen). The purity and quantity of total RNAs were evaluated by 1% formaldehyde-agarose gel electrophoresis and spectrophotometer measurement (NanoDrop 2000, Thermo).

### MicroRNA microarray assays and hierarchical cluster analysis

The RNA samples were labeled using the miRCURY™ Hy3™/Hy5™ Power labeling kit (Exiqon) and hybridized on the miRCURY™ LNA Array v.16.0 (Exiqon). Following the washing steps the slides were scanned using the Axon GenePix 4000B microarray scanner. Scanned images were then imported into GenePix Pro 6.0 software (Axon) for grid alignment and data extraction. Replicated miRNAs were averaged and miRNAs that intensities >50 in all samples were chosen for calculating normalization factor. Expressed data were normalized using the median normalization. After normalization, differentially expressed miRNAs were identified through volcano plot filtering. Hierarchical clustering showing distinguishable miRNA expression profiling among samples was performed using MEV software v4.6 (TIGR).

### Quantitative Real-time PCR

The miScript Reverse Transcription Kit and miScript SYBR Green Kit (Qiagen) were used for cDNA synthesis and SYBR Green qRT-PCR according to the manufacturer's protocol. The qRT-PCR assays were analyzed by LightCycler 480 II system (Roche). Cycling variables were set as follows: 95°C for 15 minutes, followed by 45 cycles including 95°C (15 seconds), 55°C (30 seconds) and 70°C (30 seconds). Human RNU6B snRNA was served as an internal control for RNA normalization. All reactions were carried out in triplicate.

### Construction and transfection of miR-133b precursor expression vector

The vector pEGP-MR04 for expressing miRNA precursor was purchased from GeneCopoeia. The miR-133b precursor (pre-miR-133b) was inserted into an enzyme site of the vector. An irrelevant gene was inserted into the same sites of pEGP-MR04 vector, used as a control vector (indicated as miR-null). According to the manufacturer's instructions, vectors were transfected into osteosarcoma cell lines U2-OS and MG-63 using X-tremeGENE HP DNA transfection reagent (Roche). Since the vector contains eGFP and puromycin resistance markers, successful plasmid transfection were observed by an inverted fluorescence microscope (I×70, Olympus) and all transfected cells were treated with 1 µg/ml puromycin continuously to obtain stable transfectants.

### Cell proliferation assay

U2-OS or MG-63 cells (4,000 cells per well) were seeded onto 96-well plates and incubated in corresponding medium supplemented with 10% FBS. After 24, 48, 72, and 96-hour incubation, 10 µl of CCK-8 was adding into each well, followed by four-hour incubation. Absorbance value at 450 nm was then measured. Experiments were carried out in triplicate.

### Flow cytometry assay

According to the manufacture's protocol, the apoptosis of U2-OS or MG-63 cells were measured using Guava Nexin Reagent (Millipore). 1×10^6^ cells were collected and resuspended in 100 µl medium supplemented with 10% FBS, and then incubated with 100 µl of Annexin V-PE and 7-AAD labeling solution for 20 min at room temperature. Cells were then analyzed on Guava EasyCyte 5HT flow cytometer (Millipore) using 488 nm excitation and a 575 nm bandpass filter for PE detection, and using 546 nm excitation and a 647 nm filter for 7-AAD detection. The data were analyzed by Guava Nexin Software v2.2.2.

### Boyden chamber assay

Invasion and migration of osteosarcoma cells were evaluated using the Falcon™ Cell Culture Inserts and Matrigel™ Basement Membrane Matrix (BD Bioscience). Briefly, the transwell inserts with 8 µm pores were coated with Matrigel to evaluate cell invasion, while control inserts without Matrigel were used to evaluate cell migration. 2.5×10^4^ cells were seeded onto the membrane of upper chamber in 0.5 ml of serum-free culture medium. Medium in the lower chamber contained 5% FBS was served as a chemoattractant. After 24-hour incubation, the cells on the lower surface of the membrane were stained with 0.1% crystal violet solution and photographed. The cells on the membrane were then lysed by 10% acetic acid solution and the crystal violet in the cells was released. Absorbance value at 570 nm was then measured. Assays were performed in triplicate.

### Western blotting

Total proteins were extracted by RIPA lysis buffer and protein concentration was determined by BCA protein assay (Peirce). Proteins were then separated by 10% SDS-PAGE and transferred to PVDF membranes (Millipore). Membranes were incubated in primary antibodies (1∶1000) overnight at 4°C and then in HRP-conjugated secondary antibodies (1∶5000). Signals were then visualized by ChemiDoc MP Imaging System (Bio-Rad).

### Statistical analysis

Results are presented as means ± S.E.M. Data were analyzed by SPSS v19.0, and curves and histograms were drawn using GraphPad PRISM v5.0. Statistical comparisons between two groups in qRT-PCR, cell proliferation data were analyzed using Student's t-test, whereas the comparisons among groups in apoptosis, invasion and migration assays were evaluated by One-way ANOVA and Post Hoc multiple comparison LSD. The difference was considered to be significant at *p*≤0.05.

## Results

### Identification of differentially expressed miRNAs in human osteosarcoma samples

**Figure 1:**
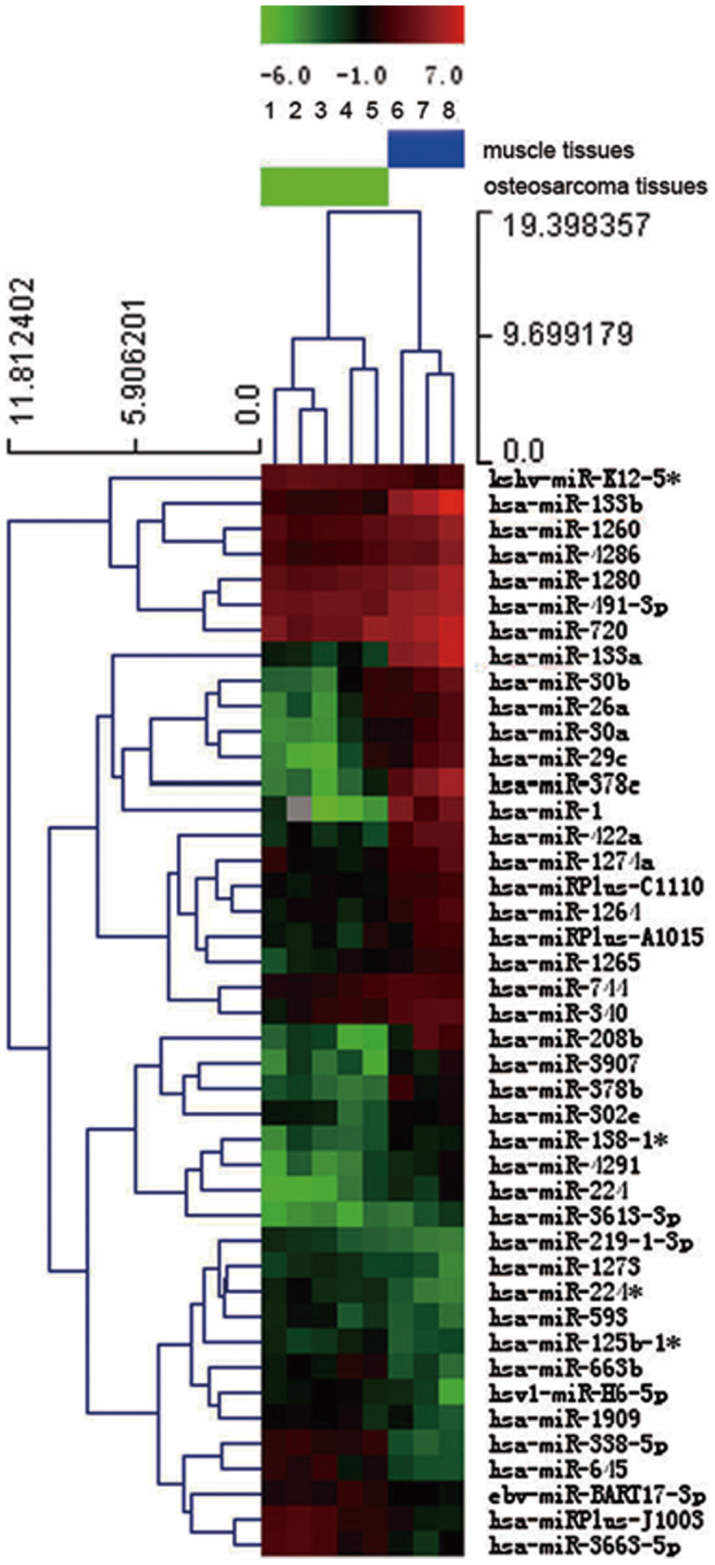
Hierarchical cluster analysis of differentially expressed miRNAs in human osteosarcoma samples. All of the miRNAs meet the criteria of volcano plot filtering (fold change ≥2.0 and *p*≤0.05). Red denotes high expression levels, whereas green depicts low-expression levels. Each listed miRNA is significantly differentially expressed (*p*≤0.05) between the osteosarcoma and normal muscle samples.

**Figure 2:**
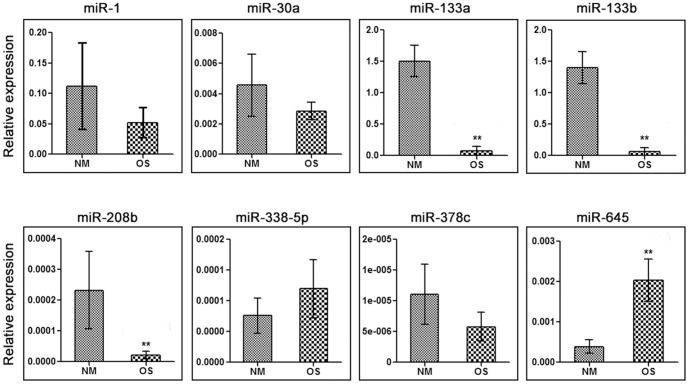
Relative expression of top eight differentially expressed miRNAs in human osteosarcoma samples (OS) using SYBR Green qRT-PCR. Human normal muscle (NM) RNAs were used as controls. The average expression levels of miR-133a, miR-133b and miR-208b in osteosarcoma tissues were significantly down-regulated, while the average miR-645 expression was significantly up-regulated (^**^, *p*≤0.01).

The thresholds of volcano plot filtering used to screen differentially expressed miRNAs are fold change ≥2.0 and *P* value≤0.05. In total, 43 miRNAs showed significant differential expression, among which 29 miRNAs were down-regulated and 14 miRNAs were up-regulated (Figure 1). The top down-regulated miRNAs (miR-1, miR-30a, miR-133a, miR-133b, miR-208b and miR-378c) and up-regulated miRNAs (miR-338-5p, miR-663b, miR-645 and miR-3663-5p) are listed in Table 2 and 3. To confirm the results of miRNA microarray assay, SYBR Green qRT-PCR was performed using the RNAs from five human osteosarcoma and three normal muscle samples in miRNA microarray assay as templates. We found that miR-133a, miR-133b and miR-208b expressions significantly decreased in osteosarcomas (*p*≤0.01) while miR-645 expression significantly increased (*p*≤0.01) (Figure 2).

**Table 2:** Normalized expression of six most significantly decreased miRNAs in human osteosarcomas.

Name	OS1	OS2	OS3	OS4	OS5	NM1	NM2	NM3	Average	Average	Fold change	*P*-value
									OS	NM		
hsa-miR-1	0.015*	0.283	N/A	0.024	0.051	15.234	11.239	2.534	0.093	9.669	103.67	0.0284
hsa-miR-30a	0.053	0.056	0.066	0.257	0.733	0.744	3.935	1.841	0.233	2.173	9.318	0.0333
hsa-miR-133a	0.176	0.38	0.325	0.453	0.202	18.069	81.693	24.533	0.307	41.432	134.777	0.0318
hsa-miR-133b	1.301	1.863	1.306	1.63	0.993	20.165	132.021	48.648	1.4189	66.944	47.181	0.0369
hsa-miR-208b	0.189	0.108	0.232	0.035	0.041	0.356	2.087	4.352	0.121	2.265	18.706	0.0445
hsa-miR-378c	0.032	0.073	0.1	0.1	0.365	3.882	37.079	14.176	0.134	18.379	137.023	0.0437

Abbreviations: OS, osteosarcoma; NM, normal muscle; N/A, not available.

, The number is the normalized expression of the microRNA, which reflects the relative abundance of miRNA in the samples.

**Table 3:** Normalized expression of four most significantly increased miRNAs in human osteosarcomas.

Name	OS1	OS2	OS3	OS4	OS5	NM1	NM2	NM3	Average	Average	Fold change	*P*-value
									OS	NM		
hsa-miR-338-5p	0.963*	1.236	1.55	0.939	1.401	0.151	0.138	0.107	1.218	0.132	0.108	0.0005
hsa-miR-663b	0.409	0.391	0.512	1.054	0.836	0.137	0.097	0.23	0.64	0.155	0.241	0.0333
hsa-miR-645	1.589	1.293	0.924	0.41	0.777	0.209	0.142	0.144	0.999	0.165	0.165	0.0224
hsa-miR-3663-5p	2.623	2.995	3.581	0.823	1.245	0.429	0.528	0.254	2.254	0.404	0.179	0.0389

Abbreviations: OS, osteosarcoma; NM, normal muscle.

, The number is the normalized expression of the microRNA, which reflects the relative abundance of miRNA in the samples.

### MiR-133b expression is decreased in paraffin-embedded osteosarcoma samples

**Figure 3:**
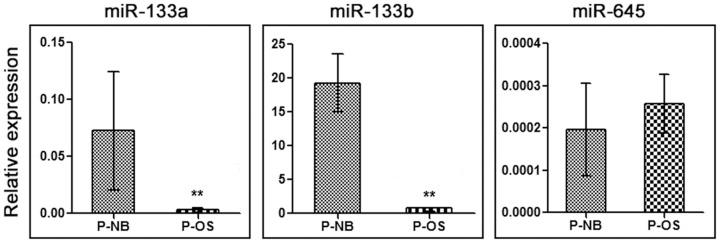
Confirmation of miR-133a, miR-133b and miR-645 expression in paraffin-embedded human osteosarcoma samples (P-OS) using SYBR Green qRT-PCR. Paraffin-embedded human normal bone (P-NB) RNAs were used as controls. The decreased expression of miR-133a and miR-133b in paraffin-embedded osteosarcoma was consistent with those in frozen osteosarcoma samples (^**^, *p*≤0.01), whereas the increased expression of miR-645 was not obviously observed (*p*≥0.05).

For further validation of our microarray results, eighteen paraffin-embedded human osteosarcoma samples and two normal bones obtained from patients P-OS-4 and P-OS-10 were employed to evaluate the expression level of miR-133a, miR-133b and miR-645 by qRT-PCR. Results showed that compared with normal bones, miR-133a and miR-133b expression were significantly down-regulated in paraffin-embedded osteosarcomas (*p*≤0.01), being consistent with results in frozen osteosarcoma samples. However, miR-645 expression didn't increase consistently in paraffin-embedded osteosarcomas (*p*>0.05) (Figure 3).

### MiR-133b inhibits osteosarcoma cell proliferation and induces apoptosis

We firstly over-expressed the miR-133b in U2-OS and MG-63 cells, and stable tranfectants were selected by puromycin. Confluent monolayer OS cells with positive green fluorescence were observed in stable transfectants of either pre-miR-133b vector or control vector (Figure S1 in File S1). The qRT-PCR results showed that compared with non-treated OS cells and OS cells transfected with control vector miR-null, miR-133b was significantly over-expressed in U2-OS and MG-63 cells with stable pre-miR-133b expression (Figure S1 in File S1).

**Figure 4:**
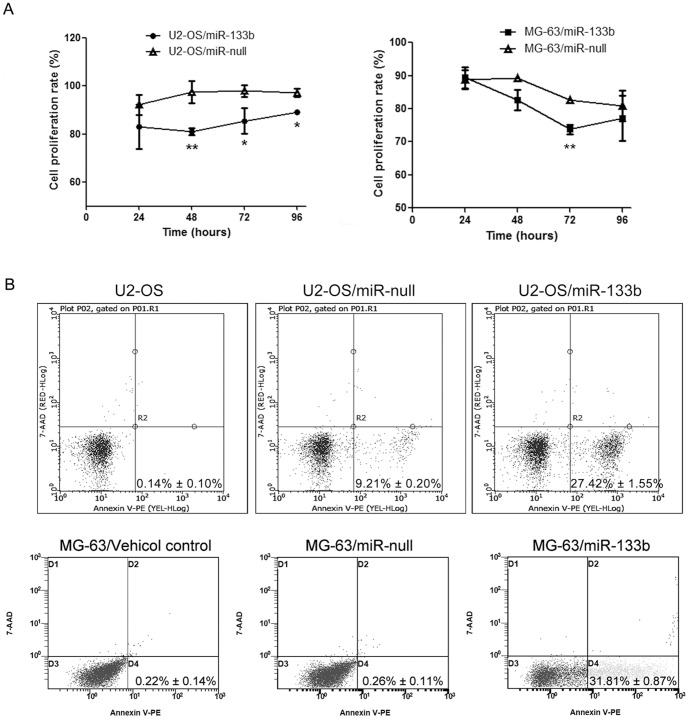
Roles of miR-133b in cell proliferation and apoptosis of osteosarcoma cell lines. (A) Over-expression of miR-133b in osteosarcoma cell lines U2-OS and MG-63 inhibited cell proliferation. U2-OS or MG-63 cells were transfected with either miR-133b precursor expression vector or pEGP-miR-null control vector, and stable clones were selected with puromycin. Compared with cells expressing miR-null, U2-OS cells with stable miR-133b expression exhibited decreased cell proliferation in 48, 72 and 96-hour incubation, while cell proliferation of MG-63 cells with stable miR-133b expression was decreased in 72 hours. Proliferation rate was normalized with absorbance value of non-treated U2-OS or MG-63 cells (^*^, *p*≤0.05; ^**^, *p*≤0.01). (B) Over-expression of miR-133b in osteosarcoma cell lines U2-OS and MG-63 induced apoptosis. Cells were stained with Annexin V-PE and 7-AAD. Data were shown as mean ± SEM. (n = 3) and were representative of one of three independent experiments.

To determine the phenotype of miR-133b over-expression in the growth and survival of osteosarcoma cell lines U2-OS and MG-63, cell proliferation and apoptosis were evaluated among miR-133b stably-expressed cells, non-treated cells and cells transfected with control vector. After 48, 72 and 96-hour incubation, osteosarcoma cells U2-OS with stable miR-133b expression exhibited decreased cell proliferation (*p*≤0.05), whereas MG-63 cells over-expressed miR-133b displayed reduced cell proliferation in 72-hours incubation (*p*≤0.05) (Figure 4A). In addition, U2-OS and MG-63 cells with stable miR-133b expression displayed higher early apoptosis (Figure 4B). 27.42% of U2-OS cells with stable miR-133b expression showed apoptosis, whereas only 9.21% of U2-OS cells with miR-null expression underwent apoptosis (*p*≤0.05). 31.81% of MG-63 cells with miR-133b over-expression displayed apoptosis, whereas only 0.26% of MG-63 cells with miR-null over-expression underwent apoptosis (*p*≤0.05).

### MiR-133b suppresses osteosarcoma cells invasion and migration

**Figure 5:**
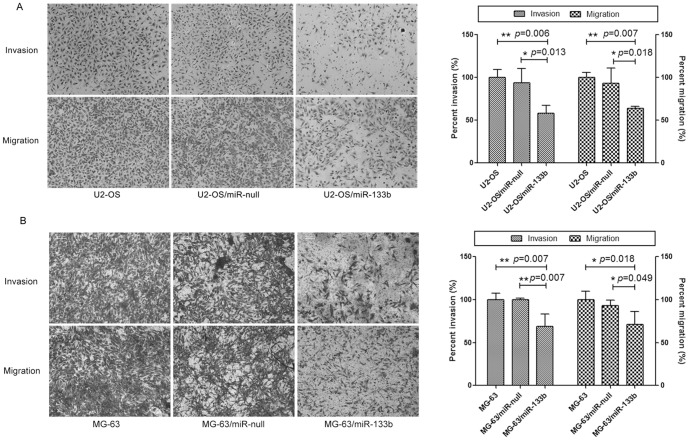
Roles of miR-133b in invasion and migration of osteosarcoma cell lines. (A) and (B), over-expression of miR-133b suppressed cell invasion and migration in U2-OS and MG-63 cells. Cell invasion and migration were visualized and examined by inverted phase contrast microscope (left, A and B; magnification: ×100). Compared to non-treated U2-OS cells or cells with miR-null expression, both percent invasion and percent migration of miR-133b over-expressed U2-OS and MG-63 cells were significantly decreased (^*^, *p*≤0.05; ^**^, *p*≤0.01). The data were representative of one of three independent experiments.

The percent invasion is represented as the ratio of cells invading through Matrigel insert membrane over cells migrating through the control membrane, while the percent migration stands for the ratio of test cells in comparison to the control cells (non-treated U2-OS) migrating through the membrane. We found that both invading and migrating miR-133b over-expressed U2-OS and MG-63 cells greatly decreased (Figure 5, left), and both percent invasion and percent migration of miR-133b over-expressed OS cells were also significantly decreased in comparison to those of miR-null over-expressed cells (*p*≤0.05) (Figure 5, right).

### MiR-133b down-regulates the expression of BCL2L2, MCL-1, IGF1R, MET, phospho-Akt and FAK

**Figure 6:**
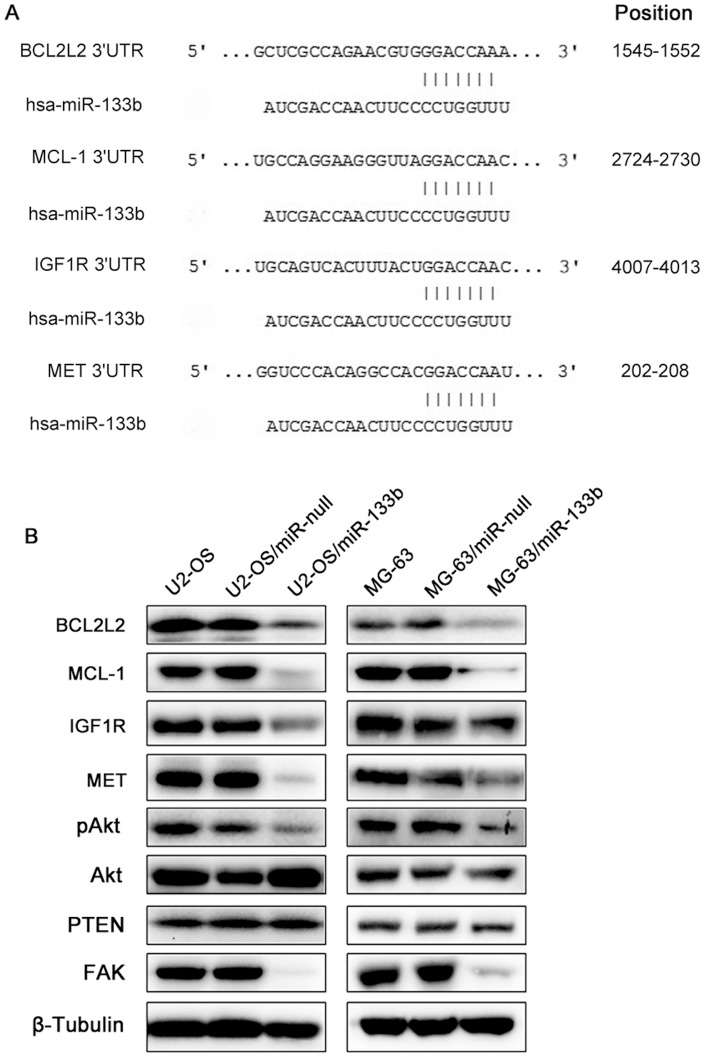
MiR-133b decreases the expression of BCL2L2, MCL-1, IGF1R, MET, phospho-Akt and FAK. (A) BCL2L2, MCL-1, IGF1R and MET are predicted as the target genes of miR-133b. Location of predicted 3′UTR target sites for miR-133b in BCL2L2, MCL-1, IGF1R and MET is presented based on TargetScan 6.2. (B) Over-expression of miR-133b decreased the expression of BCL2L2, MCL-1, IGF1R, MET and FAK, and inhibited Akt phosphorylation in U2-OS and MG-63 cells. Expression of β-Tubulin was used as a control.

To investigate the effect of miR-133b over-expression on the expression of target genes in osteosarcoma cells, as well as to study the molecular mechanism how miR-133b influences the phenotype of osteosarcoma cell, we measured the expression of BCL2L2, MCL-1, IGF1R, MET, phospho-Akt, PTEN and FAK using Western blotting. BCL2L2, MCL-1, IGF1R and MET were predicted as the target genes of miR-133b through TargetScan database (Figure 6A). The western blotting results showed that over-expression of miR-133b decreased the expression of BCL2L2, MCL-1, IGF1R and MET in osteosarcoma cells U2-OS and MG-63 (Figure 6B). Furthermore, the expression of phospho-Akt and FAK was also reduced in miR-133b over-expressed cells (Figure 6B).

## Discussion

MicroRNAs can act as oncogenes or tumor suppressor genes to mediate cancer growth, development and progression. To date, expression profiles of miRNAs in osteosarcoma are identified [14]–[18]. Duan et al. found 26 differentially expressed miRNAs in osteosarcoma cell lines compared with normal human osteoblasts [14]. Maire et al. identified 38 differentially expressed miRNAs from seven human osteosarcoma tissue samples compared with normal human osteoblasts [15]. Novello et al. found that 12 miRNAs including miR-1, miR-133b and miR-378 were differentially expressed in high-grade and low-grade OS [18]. A miRNA expression profile of osteosarcoma cell lines and tumor samples was also identified by qRT-PCR array recently and 22 differentially expressed miRNAs were found [16]. However, these studies rarely share the common miRNAs differentially expressed. Moreover, even in the same study, several differentially expressed miRNAs in osteosarcoma tissues do not consistently expressed in osteosarcoma cell lines [16]. It indicates that the difference among the sources of cell lines, tissues or patients may be able to affect the miRNA expression profile of osteosarcoma.

In this study, we identified 43 differentially expressed miRNAs in human osteosarcoma samples compared with normal skeletal muscle. Considering it is difficult to obtain the normal bone samples, normal skeletal muscle is also a suitable negative control for osteosarcoma, which have been used previously as controls for miRNA expression studies in sarcoma including osteosarcoma [8], [14]. In addition, no correlation between the histological subtypes of osteosarcoma and the expression of miR-133a or miR-133b was found in our study. Jones and his colleagues identified the miRNA expression profile of conventional, chondroblastic, and telangiectatic osteosarcoma [19]. Although these three subtypes of osteosarcoma have distinct pathological features, they share a common miRNA expression pattern, indicating that the histological subtypes can't influence the miRNA expression profile of osteosarcoma. Therefore, it is not feasible to identify different histological subtypes of osteosarcoma by miRNA profiling.

Among the 43 differentially expressed miRNAs, a part of miRNAs such as miR-1, miR-26a, miR-30a, miR-30b miR-133a, miR-133b and miR-224, are found to play a key role in cancers, whereas some miRNAs are not reported [20]–[26]. Decreased expression of miR-1 and miR-133a are found in bladder cancer, and over-expression of miR-1 or miR-133a inhibits bladder cancer cell proliferation, migration and invasion, and increases apoptosis [20]. Besides, evidence reveals that other miRNAs such as miR-21, miR-34a, miR-143 and miR-199a-3p not found in our study are also involved in the regulation of osteosarcoma growth and metastasis [14], [27]–[29]. It is reported that miR-21 is aberrantly over-expressed in osteosarcoma and knockdown of miR-21 inhibits cell invasion and migration in osteosarcoma cell line MG-63 [29]. In addition, miR-199a-3p is significantly decreased in osteosarcoma tissue samples and cell lines U2-OS and KHOS. Ectopic over-expression of miR-199a-3p in osteosarcoma cell lines decreases cell proliferation and migration which is associated with a increase of G_1_-phase and a decrease of the S-phase cell population [14].

In this study, the expression levels of miR-133a and miR-133b were confirmed to be reduced significantly in both frozen and paraffin-embedded osteosarcoma samples. A study also identifies that miR-1/miR-133a and miR-206/miR-133b clusters are down-regulated in several osteosarcoma cell lines compared with normal bones, which is consistent with our findings [17]. We also found that the expression level of miR-133b in paraffin-embedded normal bones was higher than those of miR-133a (Figure 3). The average relative expression level of miR-133b in paraffin-embedded normal bones was 19.29, whereas the average relative expression level of miR-133a in paraffin-embedded normal bones was only 0.07. Therefore, to increase the chance to get the meaningful results in functional studies, we focused on miR-133b in the following studies. We also performed pilot studies using miR-133a mimic in osteosarcoma cell lines U2-OS and MG-63. Although miR-133a and miR-133b locate on the different chromosomal regions of human genomes, they share several target genes such as BCL2L2, IGF1R MCL-1 and MET, since they are only distinguished by a single nucleotide at the 3′-end. However, results showed that the miR-133a mimic did not significantly decrease cell proliferation and migration of OS cells as miR-133b (Figure S3 in File S1). It only induced moderate apoptosis of U2-OS cells, whereas has no effect on apoptosis of MG-63 cells (Figure S4 in File S1). Western blotting results also showed that transfection of miR-133a mimic decreased the expression of MCL-1 but not BCL2L2 in U2-OS cells (Figure S5 in File S1). Evidence shows that BCL-xL and MCL-1 are targets of miR-133a, and over-expression of miR-133a inhibits cell proliferation and promotes cell apoptosis of osteosarcoma cell lines through decreasing BCL-xL and MCL-1 expression [30]. It indicates that miR-133a mimic may promote apoptosis of U2-OS cells through suppressing MCl-1 expression in our study. However, we did not find decreased MCL-1 expression and increased apoptosis in MG-63 cells. We observed that miR-133a mimic is more difficult to transfect into MG-63 cells than U2-OS cells, and the relative expression of miR-133a in MG-63 cells after miR-133a mimic transfection is lower than those in U2-OS cells (Figure S2 in File S1). Therefore, the amount of miR-133a in MG-63 cells may not be enough to decrease MCL-1 expression and induce apoptosis. As described above, compared with miR-133a, miR-133b is a more promising candidate to develop novel targets of osteosarcoma therapy.

Evidence reveals that miR-133b is specifically expressed in muscle tissues and plays an important role in muscle development, myocardial differentiation and cardiac hypertrophy [7], [31]–[34]. Studies also show that miR-133b is significantly down-regulated in several cancers such as gastric cancer, colorectal cancer, bladder cancer, prostate cancer and lung cancer [9]–[13], [35]. The miR-133b targets epidermal growth factor receptor (EGFR) and ectopic expression of miR-133b inhibits cell proliferation, migration and invasion in prostate cancer cell lines [13]. However, the biological roles of miR-133b in osteosarcoma growth and invasion are not clear yet. We found that over-expression of miR-133b in osteosarcoma cell lines U2-OS and MG-63 suppressed cell proliferation, migration and invasion, and induced apoptosis. Similar results are also reported in Novello's study, which demonstrates the decreased miR-1 and miR-133b expression in osteosarcoma cell lines and clinical samples [18]. In addition, over-expression of miR-1 and miR-133b inhibits osteosarcoma cell proliferation and invasion through cell cycle arrest and decreasing MET expression [18]. These findings indicate that miR-133b is down-regulated in several tumors including osteosarcoma, acting as a tumor suppressor gene in osteosarcoma by regulating survival, cell cycle, cell proliferation, invasion and migration.

Further, to better understand the mechanisms how miR-133b regulates cellular phenotypes of osteosarcoma cells, we first measured the expression of BCL2L2, MCL-1, IGF1R and MET, which are identified as the target genes of miR-133b in several cancers [12], [36], [37]. Further, the expression levels of phospho-Akt, PTEN and FAK were also measured. Results showed that expression of BCL2L2, MCL-1, IGF1R, MET, phospho-Akt and FAK were significantly decreased in miR-133b over-expressed osteosarcoma cell lines U2-OS and MG-63. The decrease expression of anti-apoptotic molecules BCL2L2 (Bcl-w) and MCL-1 may contributes to the increasing apoptosis of osteosarcoma cells. Both BCL2L2 and MCL-1 are members of the Bcl-2 family, which are able to promote cells survival. BCL2L2 can form Bax/Bcl-w and Bak/Bcl-w complexes by binding to pro-apoptotic protein Bax and Bak. Down-regulation of Bcl-w activates Bax and Bak, and then induces apoptosis [38], [39]. Further, MCL-1 can arrest pro-apoptotic protein Bak and form a heterodimer with Bak through the BH3 domain. Then Bak is inactivated and inhibits the activation of caspase cascade to promote cell viability [40]. Evidence shows that over-expression of miR-133b also reduces the expressions of both BCL2L2 and MCL-1 and induces apoptosis in lung cancer cell lines [12]. In addition, miR-133b over-expression in OS cells also decreased the expression of IGF1R and MET, which are the members of receptor tyrosine kinases (RTKs), leading to the decreased phosphorylation of Akt. RTKs undergo conformational alterations and autophosphorylation and then activate PI3K/Akt signaling. Activation of Akt, in turn, phosphorylates downstream pathways to mediate cell growth, motility and apoptosis [41]. Moreover, IGF1R play important roles in tumor growth and metastasis of osteosarcoma. Evidence shows high expression of IGF1R contributes to osteosarcoma cell growth, invasion and migration, and is correlated with poor prognosis of OS patients [42], [43]. Knockdown of IGF1R in osteosarcoma cell lines induces apoptosis, inhibits cell proliferation and enhances chemo- and radio-sensitivities by decreasing Bcl-2 expression and increasing Caspase-3 and Bax expression [44], [45]. Focal adhesion kinase (FAK) is found to be associated with cell migration, invasion and adhesion, through interacting with integrin and growth factors and then activating several cellular signaling pathways [46]. Therefore, decreased FAK expression may contribute to osteosarcoma cells invasion and migration. In addition, ectopic expression of miR-133b in colorectal cancer not only decreases MET expression, but also inhibits cell proliferation and induces apoptosis by G_1_-phase arrest *in vitro* and *in vivo* [36]. These indicate that miR-133b play a role as a tumor suppressor gene in osteosarcoma through inhibiting PI3K/Akt signaling and down-regulating several anti-apoptotic molecules and oncogenes such as BCL2L2, MCL-1, IGF1R, MET, and FAK.

In summary, miR-133b expression is significantly decreased in human osteosarcoma samples and is a potential tumor suppressor gene. Over-expression of miR-133b in osteosarcoma cell lines U2-OS and MG-63 decreases the expression of BCL2L2, MCL-1, IGF1R, MET and FAK, leading to the inactivation of Akt. Down-regulation of those genes leads to inhibition of cell proliferation, migration and invasion, and inducing apoptosis *in vitro*. We provide a new insight in molecular therapy of osteosarcoma by over-expressing miR-133b expression, since miR-133b exhibits potent tumor suppressive activities. MiR-133b may be regarded as a promising biomarker and gene therapy target for osteosarcoma treatment.

## Supporting Information

File S1 **Figure S1. Over-expression of miR-133b in osteosarcoma cell lines U2-OS and MG-63.** (A) and (B) Stable over-expression of miR-133b in U2-OS and MG-63 cells. The pEGP-miR-133b vector and pEGP-miR-null control vector were transfected to osteosarcoma cells and stable clones were selected by puromycin. Cells with positive miR-133b expression were visualized and examined by the fluorescence microscope after 48-hour incubation (Left; A, magnification: ×40; B, magnification: ×100). Relative expression of miR-133b in osteosarcoma cells U2-OS and MG-63 was evaluated by qRT-PCR with total RNAs isolated from the indicated cells (n = 3; ^**^, *p*≤0.01). **Figure S2. MiR-133a mimic transfection in osteosarcoma cell lines U2-OS and MG-63.** U2-OS or MG-63 cells were transfected with miR-133a mimic or miRNA mimic negative control (NC) at a final concentration of 50 nM. Cells were harvested after 48 hours and total RNAs were extracted. Relative expression of miR-133a in osteosarcoma cells U2-OS and MG-63 was then evaluated by SYBR Green qRT-PCR as described in Materials and Methods (n = 3; ^**^, *p*≤0.01). **Figure S3. Effect of miR-133a mimic on OS cell proliferation and migration.** (A) Cells were transfected with miR-133a mimic or miRNA mimic negative control (NC) at a final concentration of 50 nM. And then cell proliferation was evaluated in the indicated time points of post-transfection by CCK-8 assay. Proliferation rate was normalized with absorbance value of non-treated U2-OS or MG-63 cells. (B) Cell migration assay was performed using Boyden chambers as described in Materials and Methods. Cells were transfected as in (A) and seeded into transwell inserts in 48-hours post-transfection (WT, wild type). Assays were performed in triplicate. **Figure S4. Effect of miR-133a mimic on apoptosis of OS cells.** Osteosarcoma cell lines U2-OS (A) and MG-63 (B) were transfected with miR-133a mimic or miRNA mimic negative control (NC) at a final concentration of 50 nM. And cells were stained with Annexin V-PE and 7-AAD in 48-hours post-transfection. Apoptosis was analyzed by flow cytometer as described in Materials and Methods. Compared with cells transfected with miRNA mimic negative control, 7.79% of U2-OS cells transfected with miR-133a mimic displayed apoptosis and cell death (*p*≤0.01), whereas only 0.35% of MG-63 cells transfected with miR-133a mimic showed apoptosis and cell death (*p*≥0.05). **Figure S5. MiR-133a mimic decreased the expression of MCL-1 in U2-OS cells.** Osteosarcoma cell lines U2-OS (A) and MG-63 (B) were transfected with miR-133a mimic or miRNA mimic negative control (NC) at a final concentration of 50 nM. Cells were harvested in 48-hours post-transfection and protein expression level was measured by western blotting. Expression of β-Tubulin was used as a control.
(PDF)
