# Supplementary figures and images for: MiR-133b Is Down-Regulated in Human Osteosarcoma and Inhibits Osteosarcoma Cells Proliferation, Migration and Invasion, and Promotes Apoptosis
Source: PLoS One. 2013 Dec 31;8(12):e83571. doi: 10.1371/journal.pone.0083571 (PMC3877051; doi:10.1371/journal.pone.0083571)

**Figure S1**

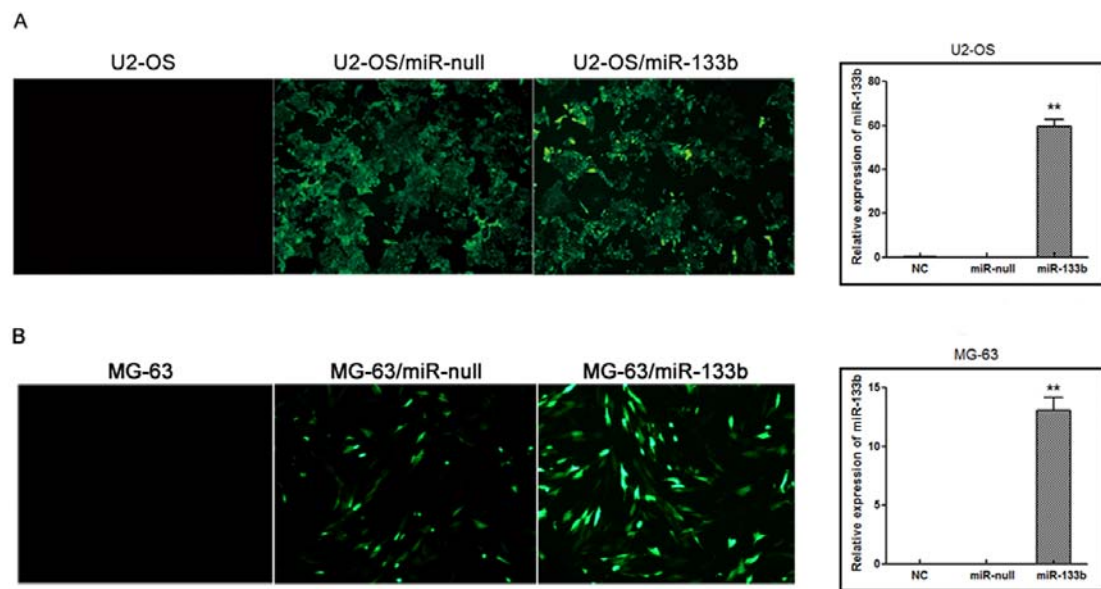

**Figure S2**

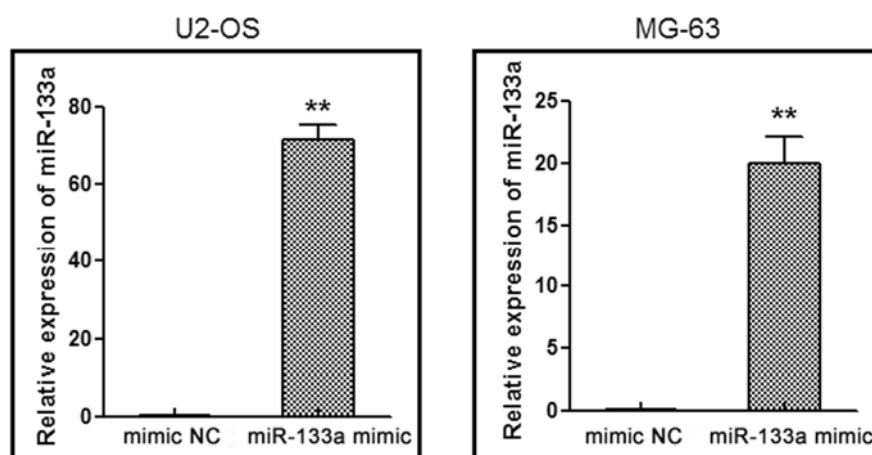

**Figure S3**

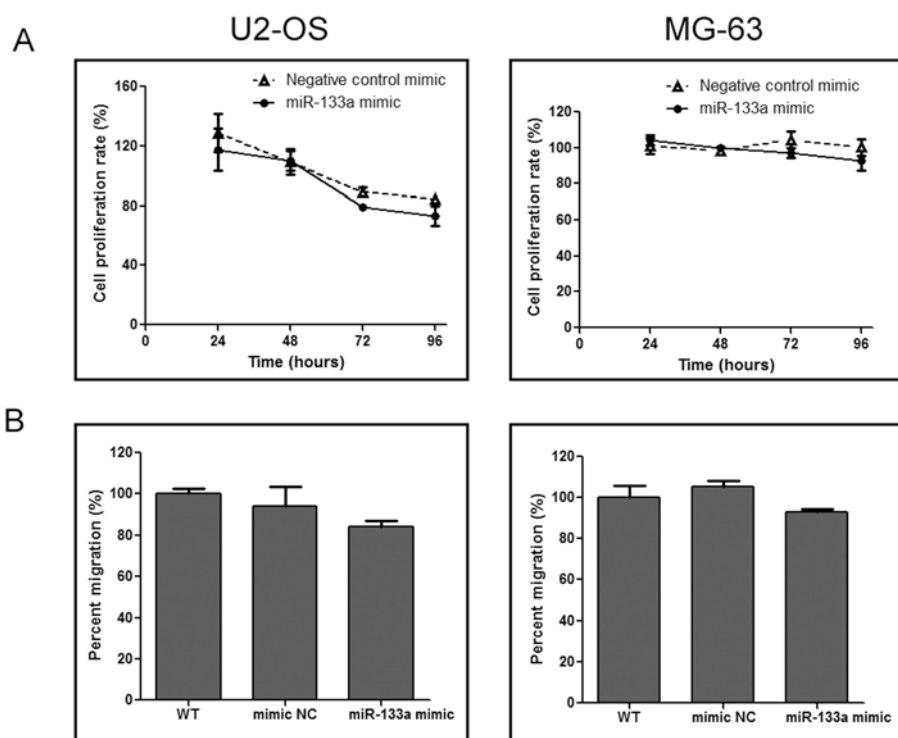

**Figure S4**

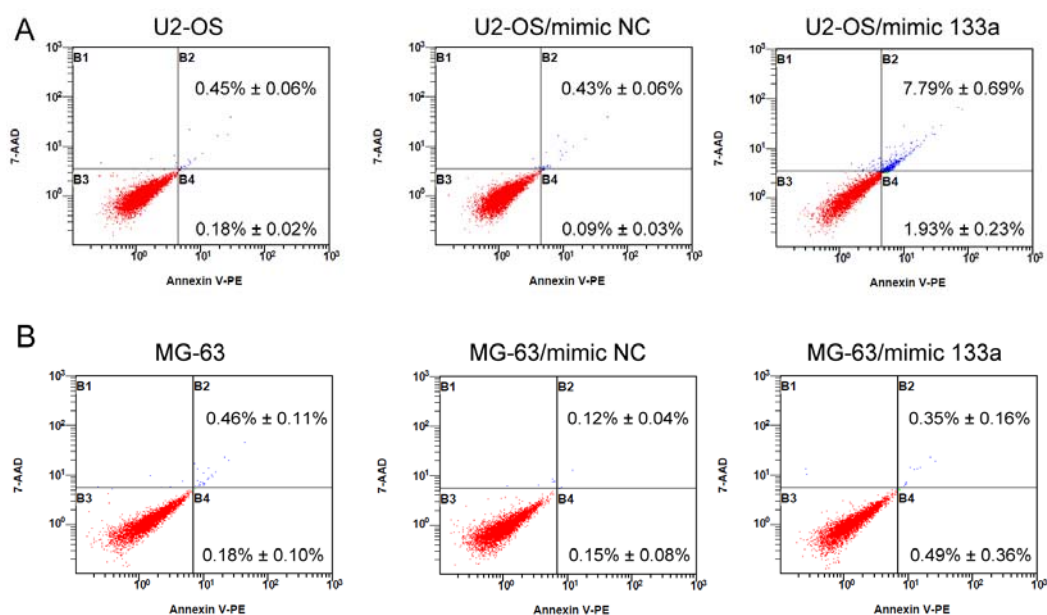

**Figure S5**

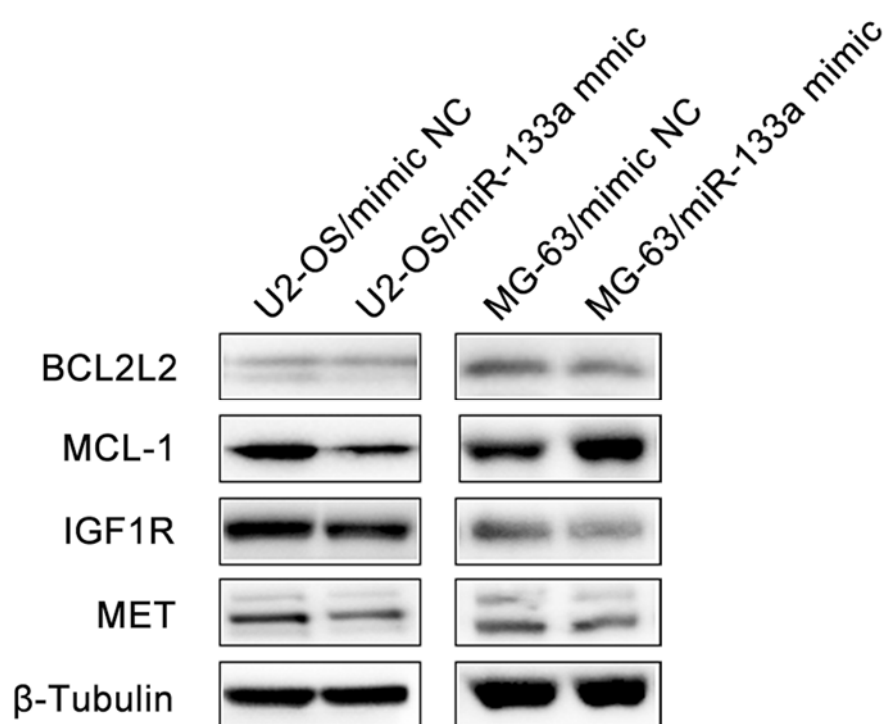

Supplement: File S1 — Figure S1. Over-expression of miR-133b in osteosarcoma cell lines U2-OS and MG-63. (A) and (B) Stable over-expression of miR-133b in U2-OS and MG-63 cells. The pEGP-miR-133b vector and pEGP-miR-null control vector were transfected to osteosarcoma cells and stable clones were selected by puromycin. Cells with positive miR-133b expression were visualized and examined by the fluorescence microscope after 48-hour incubation (Left; A, magnification: ×40; B, magnification: ×100). Relative expression of miR-133b in osteosarcoma cells U2-OS and MG-63 was evaluated by qRT-PCR with total RNAs isolated from the indicated cells (n = 3; **, p≤0.01). Figure S2. MiR-133a mimic transfection in osteosarcoma cell lines U2-OS and MG-63. U2-OS or MG-63 cells were transfected with miR-133a mimic or miRNA mimic negative control (NC) at a final concentration of 50 nM. Cells were harvested after 48 hours and total RNAs were extracted. Relative expression of miR-133a in osteosarcoma cells U2-OS and MG-63 was then evaluated by SYBR Green qRT-PCR as described in Materials and Methods (n = 3; **, p≤0.01). Figure S3. Effect of miR-133a mimic on OS cell proliferation and migration. (A) Cells were transfected with miR-133a mimic or miRNA mimic negative control (NC) at a final concentration of 50 nM. And then cell proliferation was evaluated in the indicated time points of post-transfection by CCK-8 assay. Proliferation rate was normalized with absorbance value of non-treated U2-OS or MG-63 cells. (B) Cell migration assay was performed using Boyden chambers as described in Materials and Methods. Cells were transfected as in (A) and seeded into transwell inserts in 48-hours post-transfection (WT, wild type). Assays were performed in triplicate. Figure S4. Effect of miR-133a mimic on apoptosis of OS cells. Osteosarcoma cell lines U2-OS (A) and MG-63 (B) were transfected with miR-133a mimic or miRNA mimic negative control (NC) at a final concentration of 50 nM. And cells were stained with Annexin V-P [file pone.0083571.s001.pdf]
